# Breastfeeding and the Risk of Maternal Cardiovascular Disease: A Prospective Study of 300 000 Chinese Women

**DOI:** 10.1161/JAHA.117.006081

**Published:** 2017-06-21

**Authors:** Sanne A. E. Peters, Ling Yang, Yu Guo, Yiping Chen, Zheng Bian, Jianwei Du, Jie Yang, Shanpeng Li, Liming Li, Mark Woodward, Zhengming Chen, the China Kadoorie Biobank Collaboration Group, Junshi Chen, Rory Collins, Richard Peto, Derrick Bennett, Yumei Chang, Robert Clarke, Huaidong Du, Xuejuan Fan, Simon Gilbert, Alex Hacker, Michael Holmes, Andri Iona, Christiana Kartsonaki, Rene Kerosi, Ling Kong, Om Kurmi, Garry Lancaster, Sarah Lewington, John McDonnell, Iona Millwood, Qunhua Nie, Jayakrishnan Radhakrishnan, Sajjad Rafiq, Paul Ryder, Sam Sansome, Dan Schmidt, Paul Sherliker, Rajani Sohoni, Iain Turnbull, Robin Walters, Jenny Wang, Lin Wang, Xiaoming Yang, Ge Chen, Bingyang Han, Can Hou, Jun Lv, Pei Pei, Shuzhen Qu, Yunlong Tan, Canqing Yu, Huiyan Zhou, Zengchang Pang, Ruqin Gao, Shaojie Wang, Yongmei Liu, Ranran Du, Yajing Zang, Liang Cheng, Xiaocao Tian, Hua Zhang, Silu Lv, Junzheng Wang, Wei Hou, Jiyuan Yin, Ge Jiang, Shumei Liu, Zhigang Pang, Xue Zhou, Liqiu Yang, Hui He, Bo Yu, Yanjie Li, Huaiyi Mu, Qinai Xu, Meiling Dou, Jiaojiao Ren, Shanqing Wang, Ximin Hu, Hongmei Wang, Jinyan Chen, Yan Fu, Zhenwang Fu, Xiaohuan Wang, Hua Dong, Min Weng, Xiangyang Zheng, Yijun Li, Huimei Li, Chenglong Li, Ming Wu, Jinyi Zhou, Ran Tao, Jie Shen, Yihe Hu, Yan Lu, Yan Gao, Liangcai Ma, Renxian Zhou, Aiyu Tang, Shuo Zhang, Jianrong Jin, Zhenzhu Tang, Naying Chen, Ying Huang, Mingqiang Li, Jinhuai Meng, Rong Pan, Qilian Jiang, Jingxin Qing, Weiyuan Zhang, Yun Liu, Liuping Wei, Liyuan Zhou, Ningyu Chen, Jun Yang, Hairong Guan, Xianping Wu, Ningmei Zhang, Xiaofang Chen, Xuefeng Tang, Guojin Luo, Jianguo Li, Xiaofang Chen, Jian Wang, Jiaqiu Liu, Qiang Sun, Pengfei Ge, Xiaolan Ren, Caixia Dong, Hui Zhang, Enke Mao, Xiaoping Wang, Tao Wang, Guohua Liu, Baoyu Zhu, Gang Zhou, Shixian Feng, Liang Chang, Lei Fan, Yulian Gao, Tianyou He, Li Jiang, Huarong Sun, Pan He, Chen Hu, Qiannan Lv, Xukui Zhang, Min Yu, Ruying Hu, Le Fang, Hao Wang, Yijian Qian, Chunmei Wang, Kaixue Xie, Lingli Chen, Yaxing Pan, Dongxia Pan, Yuelong Huang, Biyun Chen, Donghui Jin, Huilin Liu, Zhongxi Fu, Qiaohua Xu, Xin Xu, Youping g, Weifang Jia, Xianzhi Li, Libo Zhang, Zhe Qiu

**Affiliations:** ^1^ George Institute for Global Health University of Oxford United Kingdom; ^2^ Medical Research Council Population Health Research Unit University of Oxford United Kingdom; ^3^ Clinical Trials Service Unit and Epidemiological Studies Unit University of Oxford United Kingdom; ^4^ Chinese Academy of Medical Sciences Dong Cheng District Beijing China; ^5^ Hainan CDC Haikou Hainan China; ^6^ Jiangsu CDC NCDs Prevention and Control Department Nanjing Jiangsu China; ^7^ Qingdao CDC Qingdao China; ^8^ Department of Public Health Beijing University Beijing China; ^9^ The George Institute for Global Health University of New South Wales Sydney Australia; ^10^ Department of Epidemiology Johns Hopkins University Baltimore MD

**Keywords:** breastfeeding, cardiovascular disease, China, epidemiology, risk factor, women, Cardiovascular Disease, Epidemiology, Women, Risk Factors

## Abstract

**Background:**

Breastfeeding confers substantial benefits to child health and has also been associated with lower risk of maternal cardiovascular diseases (CVDs) in later life. However, the evidence on the effects of CVD is still inconsistent, especially in East Asians, in whom the frequency and duration of breastfeeding significantly differ from those in the West.

**Methods and Results:**

In 2004–2008, the nationwide China Kadoorie Biobank recruited 0.5 million individuals aged 30 to 79 years from 10 diverse regions across China. During 8 years of follow‐up, 16 671 incident cases of coronary heart disease and 23 983 cases of stroke were recorded among 289 573 women without prior CVD at baseline. Cox regression yielded adjusted hazard ratios (HRs) and 95% CIs for incident CVD by breastfeeding. Overall, ≈99% of women had given birth, among whom 97% reported a history of breastfeeding, with a median duration of 12 months per child. Compared with parous women who had never breastfed, ever breastfeeding was associated with a significantly lower risk of CVD, with adjusted HRs of 0.91 (95% CI, 0.84–0.99) for coronary heart disease and 0.92 (95% CI, 0.85–0.99) for stroke. Women who had breastfed for ≥24 months had an 18% (HR, 0.82; 0.77–0.87) lower risk of coronary heart disease and a 17% (HR, 0.83; 0.79–0.87) lower risk of stroke compared with women who had never breastfed. Among women who ever breastfed, each additional 6 months of breastfeeding per child was associated with an adjusted HR of 0.96 (95% CI, 0.94–0.98) for coronary heart disease and 0.97 (95% CI, 0.96–0.98) for stroke.

**Conclusions:**

Among Chinese women, a history of breastfeeding was associated with an ≈10% lower risk of CVD in later life and the magnitude of the inverse association was stronger among those with a longer duration of breastfeeding.

**Clinical Perspective**

**What Is New?**

- While breastfeeding confers substantial benefits to child health, its effects on maternal cardiovascular health remain uncertain.
- This large prospective cohort study among nearly 300 000 women in China showed that, among parous women, a history of breastfeeding was associated with an ≈10% lower risk of several major cardiovascular diseases in later life.
- Among women who had ever breastfed, each additional 6 months of breastfeeding was associated with a further ≈3% to 4% lower cardiovascular disease risk.

**What Are the Clinical Implications?**

- If causal, these findings suggest that interventions to increase the likelihood and duration of breastfeeding could have persistent benefits to maternal cardiovascular health.

## Introduction

Pregnancy causes substantial changes to the maternal cardiometabolic system, including weight gain, increased insulin resistance, and higher levels of circulating lipids.^1^, ^2^ Such pregnancy‐related cardiometabolic changes may reverse more quickly and more completely with breastfeeding, with several studies reporting that women who breastfeed have more favorable cardiometabolic profiles compared with women who do not breastfeed.^3^, ^4^, ^5^ Longer breastfeeding duration has also been associated with a lower risk of metabolic syndrome,^6^ hypertension,^7^, ^8^ and diabetes mellitus in later life,^9^ which may lead to long‐term protective benefits for cardiometabolic diseases.

A few previous studies of mostly Western populations have assessed the association between breastfeeding duration and maternal risk of cardiovascular diseases (CVDs),^10^, ^11^, ^12^, ^13^, ^14^ but with inconclusive findings about the direction, magnitude, and shape of any putative relationship. Some of these discrepancies may be explained by methodological differences between studies, such as variation in study design, small sample sizes, different exposure definitions, or varying levels of covariate adjustment. Moreover, some studies were restricted to fatal CVD end points,^10^, ^11^, ^14^ whereas others only examined specific types of CVD,^12^, ^13^ typically coronary heart disease (CHD), without evaluating other major types of incident CVD, such as ischemic and hemorrhagic stroke.

Major differences in breastfeeding practices, between and within populations, may also explain some of the discordance in study findings.^15^ Breastfeeding initiation rates are typically considerably higher and breastfeeding duration is typically longer in women from low‐ and middle‐income countries, compared with those in high‐income countries. In China, breastfeeding is almost universal and many women continue to breastfeed their infants for long durations, particularly in older generations. While breastfeeding practices in China have changed considerably over recent decades, with declines in both the likelihood and duration of breastfeeding, they remain significantly different from those in the West.^16^ There is limited evidence about the long‐term relevance of breastfeeding on the risk of CVD in a contemporary Chinese population, overall or in different population subgroups. We examined the relationship between breastfeeding and the risk of several major CVDs among 300 000 women from the prospective China Kadoorie Biobank study.^17^

## Methods

### Study Population

Detailed information about the study design and procedures of China Kadoorie Biobank has been previously reported.^17^ Briefly, between 2004 and 2008, 302 669 women and 210 222 men aged 35 to 79 years were recruited from 5 urban and 5 rural areas of China. At the baseline study assessment clinics, trained health workers administered a laptop‐based questionnaire that covered demographic and socioeconomic status, lifestyle factors, and medical history. Detailed information on women's reproductive factors was also collected, including age at menarche; the number of pregnancies, abortions, and live births; and the total duration of any breastfeeding for each live birth. From this, the mean duration of breastfeeding per child and cumulative lifetime duration of any breastfeeding for all children were calculated for parous women. A series of physical measurements were taken using standard methods and a blood sample was collected for long‐term storage. Central ethical approvals were obtained from Oxford University and the China National Center for Disease Control and Prevention (CDC). Approvals were also obtained from institutional research boards at the local CDCs in the 10 areas. All study participants provided written informed consent.

### Follow‐Up for Morbidity and Mortality

Study participants were followed for cause‐specific morbidity and mortality through linkage with regional disease and death registers and with the national health insurance system. Causes of death were derived from official death certificates and were, where necessary, supplemented by reviews of medical records. Data linkage with health insurance agencies was performed every 6 months in each region to retrieve all hospitalized events occurring in that period for study participants. Active follow‐up was performed annually to minimize attrition. The end points for this study were incident CVD (I00–I99); CHD (I20–I25); cerebrovascular disease (henceforth, “stroke”) (I60–I69), including hemorrhagic stroke (I61) and ischemic stroke (I63); major CVDs (ie, I21–I23, I60–I61, I63–I64 [any] and I00–I20, I24–I25, I27–I59, I62, I65–I88, I95–I99 [only where fatal]); and fatal CVDs (I00–I99). Women with a self‐reported history of CHD or stroke at baseline were excluded (n=13 096), leaving 289 573 women for further analyses.

### Statistical Analyses

Baseline characteristics are presented as means (SDs) for continuous variables and as percentages for categorical variables. Cox proportional hazards models were used to estimate hazard ratios (HRs) and 95% CIs for the study end points associated with breastfeeding history, comparing parous women who had never breastfed with nulliparous women and with parous women who ever breastfed. The Cox proportional hazards assumption was checked using log cumulative hazard plots and appeared to be reasonable. For comparisons involving more than 2 groups, CIs were estimated using floating absolute risks, enabling valid comparisons between any 2 groups, even if neither is the baseline group.^18^ In analyses restricted to parous women, we obtained the HRs and CIs associated with mean duration of breastfeeding per child, comparing those who had never breastfed with those who breastfed each child for >0 to 6, 6 to 12, 12 to 18, 18 to 24, or >24 months. Among women who had ever breastfed, we estimated the HRs for each additional 6 months of breastfeeding per child. Analyses were stratified by age at risk (5‐year age groups) and area of residence (10 areas) and adjusted for highest level of education attained (none, primary, secondary, and tertiary or above), household income (<5000, 5000–19 999, and ≥20 000 yuan), smoking (current, former, and never), alcohol use (weekly, occasionally, and never), physical activity, systolic blood pressure, history of hypertension, history of diabetes mellitus, and body mass index.

Several sensitivity analyses were conducted. First, in analyses among women who had ever breastfed, we additionally adjusted for other reproductive factors, ie, age at menarche, age at first birth, and total number of miscarriages, induced abortions, and stillbirths. Second, among parous women, we obtained the HRs and CIs associated with cumulative lifetime duration of breastfeeding, with additional adjustment for the number of live births. Third, we restricted the analyses on breastfeeding duration to women who had given birth to only 1 child. Subgroup analyses were conducted to obtain the HRs associated with mean duration of breastfeeding per child, separately by study region and by birth cohort. Further subgroup analyses by study region, birth cohort, highest level of attained education, body mass index, smoking status, and history of hypertension were conducted to obtain the HRs comparing parous women who had ever breastfed with parous women who had never breastfed. Among those who had ever breastfed, we conducted similar subgroup analyses to obtain the HRs associated with each additional 6 months of breastfeeding per child. Analyses were performed using SAS version 9.3 and R version 3.1.2.

## Results

Of the 289 573 women included, the mean baseline age was 51 years and 99% reported having at least 1 live birth, among whom, 97% had ever breastfed and 91% had breastfed each child for at least 6 months. The median duration of breastfeeding per child was 12 months. Women who had breastfed for longer durations were older and were more likely to come from rural areas compared with women who had never breastfed or had breastfed for shorter durations (Table 1).

**Table 1:** Baseline Characteristics of Study Participants by Breastfeeding Duration

	Total	Nulliparous	Parous Women, by Breastfeeding Duration Per Child					
			Never	>0 to 6 mo	6 to 12 mo	12 to 18 mo	18 to 24 mo	>24 mo
No. (% rural)	289 573 (44)	3970 (37)	7785 (29)	17 780 (37)	131 546 (44)	65 685 (68)	42 728 (79)	20 079 (83)
Age, y	50.5 (10.3)	49.0 (11.7)	47.3 (9.2)	47.0 (9.3)	50.1 (10.2)	50.7 (10.4)	52.2 (10.8)	53.8 (9.7)
Education level, %								
Primary or below	56.8	39.6	26.2	34.2	51.8	59.1	74.2	79.5
Secondary or above	43.3	60.4	73.8	65.7	48.2	40.9	25.7	20.4
Household income, %								
Low	10.2	11.1	4.6	5.0	5.6	11.8	18.8	23.2
Middle	49.1	51.8	40.9	39.9	41.4	54.9	63.3	61.5
High	40.7	37.1	54.4	55.1	53.1	33.3	17.9	15.3
Current smoking, %	2.3	3.8	2.3	1.8	1.6	3.0	2.7	3.9
Regular alcohol use, %	2.1	3.2	2.9	2.6	1.7	2.5	1.9	2.4
Physical activity, MET h/d	17.2 (11.0–28.7)	15.0 (9.3–25.0)	16.6 (10.5–26.7)	18.0 (11.2–29.1)	17.3 (10.8–28.7)	17.0 (11.2–28.0)	18.4 (11.0–30.3)	16.5 (10.7–28.0)
Systolic blood pressure, mm Hg	129.4 (21.8)	126.3 (23.0)	123.3 (19.8)	123.1 (19.8)	128.2 (21.2)	130.2 (21.6)	133.3 (22.8)	135.2 (23.0)
Diastolic blood pressure, mm Hg	76.7 (10.8)	75.5 (11.2)	75.4 (10.7)	74.9 (10.5)	76.3 (10.7)	77.0 (10.8)	77.7 (11.2)	78.0 (11.3)
Body mass index, kg/m^2^	23.8 (3.4)	23.3 (3.7)	23.7 (3.4)	23.4 (3.3)	23.7 (3.4)	23.9 (3.5)	23.9 (3.5)	24.0 (3.6)
History of hypertension, %	10.2	8.8	8.6	7.7	10.7	10.4	10.0	10.4
History of diabetes mellitus, %	2.9	2.5	3.3	2.5	3.0	2.9	2.6	2.7
No. of livebirths, %								
1	35.2	0	77.3	60.6	42.8	29.8	15.0	14.4
2	31.9	0	14.4	26.1	31.7	33.9	35.3	38.1
≥3	31.5	0	8.4	13.3	25.6	36.3	49.7	47.5
Breastfeeding duration, mo								
Lifetime duration	24.0 (12.0–48.0)	NA	NA	6.0 (3.0–9.0)	20.0 (12.0–24.0)	34.0 (18.0–48.0)	48.0 (42.0–72.0)	78.0 (60.0–108.0)
Mean duration per child	12.0 (11.0–18.0)	NA	NA	5.0 (3.0–6.0)	12.0 (10.0–12.0)	15.7 (14.0–18.0)	24.0 (21.0–24.0)	30.0 (27.4–36.0)
Age at menarche, y	15.4 (2.0)	15.2 (2.6)	14.8 (1.9)	14.9 (1.9)	15.3 (1.9)	15.5 (1.9)	15.7 (2.0)	16.0 (2.0)
Age at first birth, y	23.4 (3.2)	NA	25.8 (3.8)	24.7 (3.4)	23.6 (3.2)	23.0 (3.0)	22.5 (2.9)	22.6 (2.8)
History of miscarriage, %	8.4	79.2	6.9	6.5	7.3	9.8	11.6	12.8
History of induced abortion, %	51.4	90.8	64.0	66.1	59.3	49.6	34.8	35.1
History of stillbirth, %	5.1	75.6	4.8	3.9	5.6	6.0	5.5	5.3

MET indicates metabolic equivalent; NA, not available.

Values are percentages for categorical variables, and means and SD or median and 25th and 75th percentile for continuous variables.

During a median of 8.1 years (quartile 1: 7.2; quartile 3: 9.1) of follow‐up, 49 377 incident cases of CVD were recorded, including 16 671 cases of CHD and 23 983 of stroke (14 290 ischemic and 2998 hemorrhagic stroke).

### History of Breastfeeding and CVD Risk

**Figure 1:**
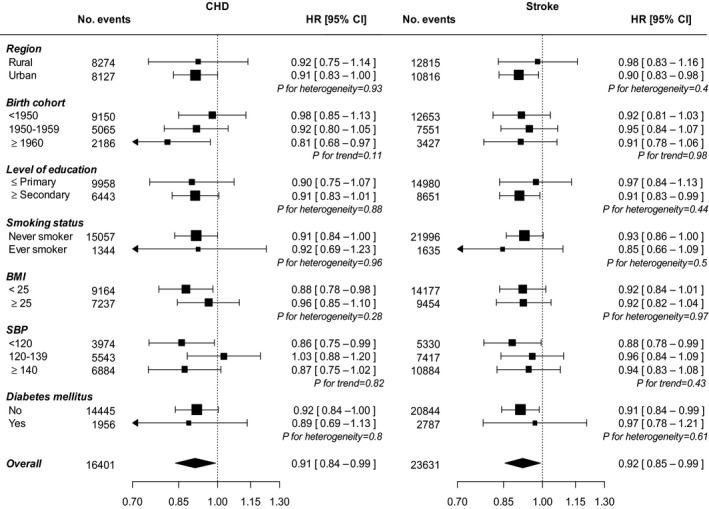
Adjusted* hazard ratios (HRs) and 95% CIs for incident coronary heart disease (CHD) and stroke comparing parous women who ever breastfed with parous women who never breastfed, by baseline characteristics. *Analyses are stratified by age at risk and study area, and, where appropriate, adjusted for level of attained education, household income, smoking status, alcohol use, systolic blood pressure (SBP), history of hypertension, physical activity, body mass index (BMI), and history of diabetes mellitus. Each square represents the HR. Horizontal lines indicate the corresponding 95% CIs. The diamond indicates the overall estimate and its 95% CI. Nulliparous women are excluded.

There were no significant differences in the risk of CVD between nulliparous women and parous women who had never breastfed (Table 2). In contrast, parous women who had ever breastfed were at a significantly lower risk of major CVD and its main components (ie, CHD and stroke) than parous women who had never breastfed; the adjusted HRs were 0.88 (95% CI, 0.80–0.97) for major CVD, 0.91 (95% CI, 0.84–0.99) for CHD, 0.92 (95% CI, 0.85–0.99) for stroke, and 0.88 (95% CI, 0.79–0.97) for ischemic stroke. The HRs did not vary materially by study areas, birth cohorts, or other population subgroups (Figure 1).

**Table 2:** CVD End Points Associated With Breastfeeding^a^

	Events, No.	Nulliparous	Parous Women^b^		Each Additional 6 mo Per Child^c^
			Never Breastfed	Breastfed	
All CVD	49 377	1.02 (0.94–1.10)	1.00 (0.95–1.05)	0.96 (0.95–0.97)	0.98 (0.97–0.99)
Major CVD	19 227	0.99 (0.89–1.11)	1.00 (0.92–1.09)	0.88 (0.87–0.90)	0.97 (0.96–0.99)
Fatal CVD	3825	1.17 (0.92–1.48)	1.00 (0.77–1.29)	0.90 (0.87–0.94)	0.98 (0.95–1.01)
Coronary heart disease	16 671	1.01 (0.90–1.14)	1.00 (0.92–1.09)	0.91 (0.89–0.93)	0.96 (0.95–0.98)
Stroke	23 983	0.95 (0.86–1.06)	1.00 (0.93–1.08)	0.92 (0.90–0.93)	0.97 (0.96–0.98)
Hemorrhagic stroke	2998	0.92 (0.68–1.25)	1.00 (0.77–1.31)	0.84 (0.81–0.88)	0.99 (0.96–1.03)
Ischemic stroke	14 290	0.96 (0.85–1.10)	1.00 (0.91–1.09)	0.88 (0.86–0.90)	0.97 (0.95–0.98)

Values are adjusted hazard ratios (95% CIs).

CVD indicates cardiovascular disease.

a Analyses are stratified by age at risk and study area and adjusted for level of attained education, household income, smoking status, alcohol use, systolic blood pressure, history of hypertension, physical activity, body mass index, and history of diabetes mellitus.

b SEs were obtained using floating absolute risks.

c Among parous women who ever breastfed only.

### Breastfeeding Duration and CVD Risk

**Figure 2:**
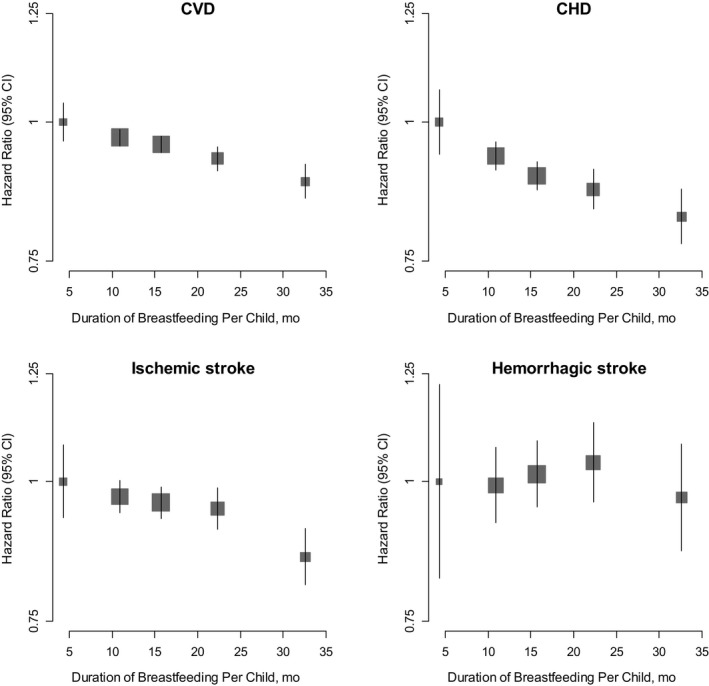
Adjusted* hazard ratios and 95% CIs for incident cardiovascular disease (CVD), coronary heart disease (CHD), ischemic stroke, and hemorrhagic stroke associated with duration of breastfeeding per child among parous women who ever breastfed. *Analyses are stratified by age at risk and study area, and adjusted for level of attained education, household income, smoking status, alcohol use, systolic blood pressure, history of hypertension, physical activity, body mass index, and history of diabetes mellitus. The hazard ratios are plotted on a floating absolute scale. Each square has an area inversely proportional to the SE of the log risk. Vertical lines indicate the corresponding 95% CIs.

**Figure 3:**
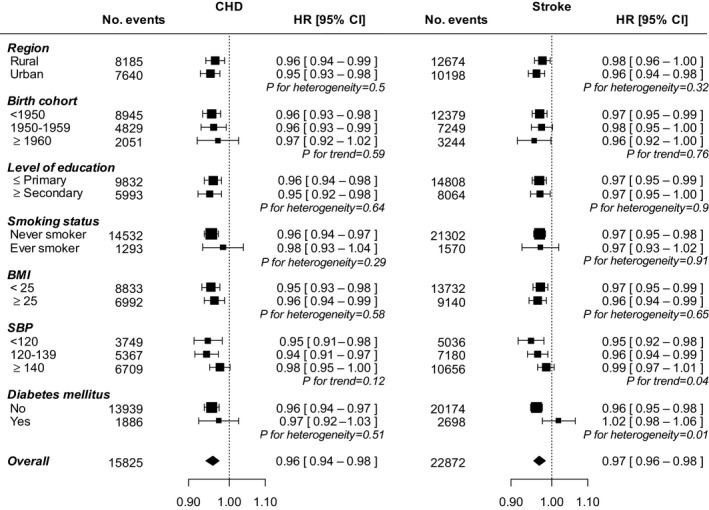
Adjusted* hazard ratios (HRs) and 95% CIs for incident coronary heart disease (CHD) and stroke associated with each additional 6 months of breastfeeding per child, by baseline characteristics. Analyses are restricted to parous women who ever breastfed. *Analyses are stratified by age at risk and study area, and, where appropriate, adjusted for level of attained education, household income, smoking status, alcohol use, systolic blood pressure, history of hypertension, physical activity, body mass index, and history of diabetes mellitus. Each square represents the HR. Horizontal lines indicate the corresponding 95% CIs. The diamond indicates the overall estimate and its 95% CI. Nulliparous women are excluded.

Among women who ever breastfed, there was an inverse log‐linear association between the duration of breastfeeding per child and the risk of several major CVDs, except hemorrhagic stroke (Figure 2 and Tables S1 and S2). Compared with women who had never breastfed, women who had breastfed between 0 to 6 months, 6 to 12 months, 12 to 18 months, 18 to 24 months, or over 24 months had a 1%, 7%, 11%, 13%, and 18% lower risk of CHD, respectively, with each additional 6 months of breastfeeding per child associated with 4% (2–5%) lower CHD risk (*P* for trend <0.001). For stroke, the association was similar, with each additional 6 months of breastfeeding per child associated with an adjusted HR of 0.97 (95% CI, 0.96–0.98) for stroke (Table 1), with limited evidence of substantial differences across population subgroups (Figure 3). Additional adjustment for women's reproductive health factors had little impact on the results (Table S1). Moreover, analyses by total lifetime duration of breastfeeding, or restricted to women with only 1 child, yielded similar associations (Tables S3 and S4). The results were broadly similar between women in urban and rural areas and did not vary by different birth cohort (Figures S1 and S2).

## Discussion

This large prospective cohort study among nearly 300 000 women in China provides the most comprehensive assessment yet of the effects of breastfeeding on the risk of major CVDs in later life. Parous women who had ever breastfed were at an ≈10% lower risk of major CVD subtypes compared with parous women who had never breastfed. Moreover, among those who had ever breastfed, each additional 6 months of breastfeeding was associated with a further ≈3% to 4% lower CVD risk. These results persisted after adjustment for a range of potential confounders and were broadly consistent across major population subgroups.

Previous studies of mostly Western populations have reported conflicting results on the relationship between breastfeeding and the risk of CVD.^10^, ^11^, ^12^, ^13^, ^14^ In the US Nurses' Health Study of 90 000 parous women with 2500 cases of CHD, there was a threshold in the association between breastfeeding and CHD; only women with a lifetime duration of breastfeeding of ≥2 years had a significantly lower risk of CHD than those who never breastfed.^13^ The HUNT trial (Nord‐Trøndelag Health Study)^14^ of 20 000 Norwegian women reported that the inverse association between a history of breastfeeding and CVD mortality was modified by age. Among women younger than 65 years, there was some evidence of a U‐shaped relationship between lifetime duration of breastfeeding and the risk of CVD death, with women who reported lifetime breastfeeding duration of 7 to 12 months having half the risk than those who had breastfed for ≥2 years. In contrast, the EPIC (European Prospective Investigation into Cancer and Nutrition)‐CVD case‐cohort study, among 15 000 women with 5000 incident cases of CHD, found an inverse association between breastfeeding and the risk of CHD at all ages.^12^ The risk of CHD was the lowest among those who had breastfed for the longest durations, suggesting that prolonged durations of breastfeeding confer additional coronary benefits. An additional study among 200 000 parous women in the EPIC trial reported that a history of breastfeeding was associated with a 20% and 31% lower risk of mortality from CVD and CHD, respectively, but did not find any significant effect of breastfeeding on stroke.^11^ In China, only 1 prospective study has previously examined the association between breastfeeding and CVD risk. In that study among 250 000 textile workers in Shanghai, born between 1925 and 1958,^10^ there were no consistent associations between breastfeeding duration and the risk of fatal CHD, ischemic stroke, or hemorrhagic stroke, which may be attributable to the relatively small number of deaths included (420 CHD, 575 ischemic stroke, and 1583 hemorrhagic stroke). In contrast, the present analysis included a substantially larger number of fatal and nonfatal CVD cases and provides the largest and most comprehensive analyses to date on the relationship between breastfeeding initiation and duration and the risk of several major CVDs among urban and rural Chinese women.

The observational nature of the present study does not allow us to determine whether the inverse relationship between breastfeeding and CVD risk is causal. However, several mechanisms have been postulated to underpin the association between breastfeeding and CVD risk in later life.^19^ Pregnancy is characterized by major changes in the maternal metabolic system to support fetal growth and in anticipation of breastfeeding, including accumulation of visceral fat, increased insulin resistance, and higher circulating lipid levels.^1^, ^2^ The “reset hypothesis” posits that breastfeeding plays a central role in mobilizing these accumulated fat stores and in resetting the maternal metabolism after birth.^3^ The longer a woman breastfeeds, the more completely these accumulated stores may be deposited, potentially with lasting benefits for maternal cardiometabolic health. Prolonged breastfeeding has been associated with improved β‐cell function,^20^ more favorable cardiovascular risk factor profiles,^4^, ^21^ and lower risks of incident hypertension and type 2 diabetes mellitus.^8^, ^9^ In contrast, studies on the potential benefits of breastfeeding on gestational weight retention and body adiposity in later life have reported mixed results.^22^, ^23^, ^24^ In a previous analysis of this study, we did not find a relationship between breastfeeding duration and general or central adiposity.^22^ Therefore, breastfeeding may also be a marker of health‐promoting behaviors, rather than a causal determinant of improved cardiometabolic outcomes itself. Several studies in Western populations have demonstrated that women who breastfeed are generally wealthier and more likely to engage in other beneficial health behaviors than women who do not breastfeed.^15^, ^25^, ^26^ However, this pattern is reversed in the present study, in which poorer women from rural areas of China were more likely to breastfeed for a longer duration than their wealthier urban counterparts. Moreover, adjustment for various sociodemographic and lifestyle factors had little impact on our findings.

The strengths of this study include its large sample size, the prospective follow‐up for the occurrence of several major CVDs, and the availability of detailed information on a range of reproductive, sociodemographic, and lifestyle‐related factors. The generalizability of the study findings was enhanced by the inclusion of women from 10 diverse localities across China. The applicability of our findings to other countries and ethnicities needs to be addressed in further research. Our study did not collect data on CVD risk factors either before or during pregnancy that might determine breastfeeding as well as future CVD risk. Therefore, we cannot preclude the possibility that the findings were subject to unmeasured or residual confounding. Moreover, our results may be liable to reverse causality as certain metabolic conditions, such as pregestational diabetes mellitus or high maternal body mass index, may affect the ability to initiate and sustain breastfeeding and may also confer a higher risk of cardiometabolic conditions.^27^, ^28^, ^29^ Furthermore, as in most previous studies, information on breastfeeding was self‐reported and solicited several years after childbirth and breastfeeding. This could have led to measurement error, which, if at random, would have underestimated the true strengths of the observed associations. Information about the duration of exclusive breastfeeding and the provision of complementary infant food was also not available, which limits interpretations of the effects seen.

Breastfeeding is one of the few beneficial health behaviors that are more common in low‐ and middle‐income countries than in high‐income countries, and within low‐ and middle‐income countries is more common among poorer than richer women.^15^ This socioeconomic gradient in breastfeeding is particularly relevant to China, a country where breastfeeding durations have declined dramatically over the past several decades and where breastfeeding practices continue to vary greatly across different parts of the country.^16^ In 2008, fewer than 16% of women in urban and ≈30% of women in rural areas exclusively breastfed their babies throughout the World Health Organization's recommended period of 6 months.^30^ Common reasons for prematurely ceasing exclusive breastfeeding are the perceived insufficiency of breast milk, traditions regarding the introduction of complementary food, and maternal or child illness. Intensive industry promotion of breast milk substitutes, limited duration of maternity leave, and inadequate facilities to continue to breastfeed at the workplace also contribute to shorter durations of breastfeeding, particularly in urban China.^31^ Although there is increasing recognition of the importance of exclusive breastfeeding, genuine commitment from policy makers is needed to implement strategies in the healthcare system, communities and families, and the work environment that promote and support every woman to breastfeed.^32^ If effective and sustained, such efforts are likely to confer major benefits to the health of children and women, along with substantial cost savings.^15^, ^32^

## Conclusions

This large prospective study showed that breastfeeding is associated with an ≈10% lower risk of several major CVDs in later life among Chinese women and the magnitude of the inverse association was stronger among those with a longer duration of breastfeeding. If causal, these findings suggest that interventions to increase the likelihood and duration of breastfeeding could have persistent benefits to maternal cardiovascular health.

## Sources of Funding

The baseline survey was funded by the Kadoorie Charitable Foundation, Hong Kong. Long‐term continuation: UK Wellcome Trust (088158/Z/09/Z, 104085/Z/14/Z), Chinese Ministry of Science and Technology (2011BAI09B01, 2012–14), Chinese National Natural Science Foundation (81390541). The British Heart Foundation, UK Medical Research Council, and Cancer Research UK provide core funding to the Oxford Clinical Trial Service Unit and Epidemiological Studies Unit. This work was also supported by grants from the National Natural Science Foundation of China (No. 81390541, No. 81390544).

## Disclosures

None.

## Supporting information


**Table S1.** Hazard Ratios (95% CIs) for Cardiovascular Disease Associated With Duration of Breastfeeding Among Parous Women Who Ever Breastfed Across Different Levels of Adjustment
**Table S2.** Hazard Ratios (95% CIs) for Cardiovascular Disease Associated With Duration of Breastfeeding Per Child Among Parous Women Across Different Levels of Adjustment, Using Those Who Never Breastfed as the Reference Group
**Table S3.** Adjusted* Hazard Ratios (95% CIs) for Cardiovascular Disease Associated With Total Lifetime Duration of Breastfeeding Among Parous Women
**Table S4.** Adjusted* Hazard Ratios (95% CIs) for Cardiovascular Disease Associated With Duration of Breastfeeding Among Women With One Livebirth, Using Those Who Never Breastfed as the Reference Group
**Figure S1.** Adjusted* hazard ratios (HRs) and 95% CIs for incident cardiovascular disease, coronary heart disease, and stroke associated with duration of breastfeeding per child among parous women who ever breastfed, by region. *Analyses are stratified by age at risk and study area and adjusted for level of attained education, household income, smoking status, alcohol use, systolic blood pressure, history of hypertension, physical activity, body mass index, and history of diabetes mellitus. The HRs are plotted on a floating absolute scale. Each square has an area inversely proportional to the SE of the log risk. Vertical lines indicate the corresponding 95% CIs.
**Figure S2.** Adjusted* hazard ratios (HRs) and 95% CIs for incident cardiovascular disease, coronary heart disease, and stroke associated with duration of breastfeeding per child among parous women who ever breastfed, by birth cohort. *Analyses are stratified by age at risk and study area and adjusted for level of attained education, household income, smoking status, alcohol use, systolic blood pressure, history of hypertension, physical activity, body mass index, and history of diabetes mellitus. The HRs are plotted on a floating absolute scale. Each square has an area inversely proportional to the SE of the log risk. Vertical lines indicate the corresponding 95% CIs.

## Members of the China Kadoorie Biobank Collaborative Group

International Steering Committee: Junshi Chen, Zhengming Chen (PI), Rory Collins, Liming Li (PI), Richard Peto. International Coordinating Centre, Oxford: Daniel Avery, Derrick Bennett, Yumei Chang, Yiping Chen, Zhengming Chen, Robert Clarke, Huaidong Du, Xuejuan Fan, Simon Gilbert, Alex Hacker, Michael Holmes, Andri Iona, Christiana Kartsonaki, Rene Kerosi, Ling Kong, Om Kurmi, Garry Lancaster, Sarah Lewington, John McDonnell, Iona Millwood, Qunhua Nie, Jayakrishnan Radhakrishnan, Sajjad Rafiq, Paul Ryder, Sam Sansome, Dan Schmidt, Paul Sherliker, Rajani Sohoni, Iain Turnbull, Robin Walters, Jenny Wang, Lin Wang, Ling Yang, Xiaoming Yang. National Coordinating Centre, Beijing: Zheng Bian, Ge Chen, Yu Guo, Bingyang Han, Can Hou, Jun Lv, Pei Pei, Shuzhen Qu, Yunlong Tan, Canqing Yu, Huiyan Zhou. 10 Regional Coordinating Centres: Qingdao Qingdao CDC: Zengchang Pang, Ruqin Gao, Shaojie Wang, Yongmei Liu, Ranran Du, Yajing Zang, Liang Cheng, Xiaocao Tian, Hua Zhang. Licang CDC: Silu Lv, Junzheng Wang, Wei Hou. Heilongjiang Provincial CDC: Jiyuan Yin, Ge Jiang, Shumei Liu, Zhigang Pang, Xue Zhou. Nangang CDC: Liqiu Yang, Hui He, Bo Yu, Yanjie Li, Huaiyi Mu, Qinai Xu, Meiling Dou, Jiaojiao Ren. Hainan Provincial CDC: Jianwei Du, Shanqing Wang, Ximin Hu, Hongmei Wang, Jinyan Chen, Yan Fu, Zhenwang Fu, Xiaohuan Wang, Hua Dong. Meilan CDC: Min Weng, Xiangyang Zheng, Yijun Li, Huimei Li, Chenglong Li. Jiangsu Provincial CDC: Ming Wu, Jinyi Zhou, Ran Tao, Jie Yang. Suzhou CDC: Jie Shen, Yihe Hu, Yan Lu, Yan Gao, Liangcai Ma, Renxian Zhou, Aiyu Tang, Shuo Zhang, Jianrong Jin. Guangxi Provincial CDC: Zhenzhu Tang, Naying Chen, Ying Huang. Liuzhou CDC: Mingqiang Li, Jinhuai Meng, Rong Pan, Qilian Jiang, Jingxin Qing, Weiyuan Zhang, Yun Liu, Liuping Wei, Liyuan Zhou, Ningyu Chen, Jun Yang, Hairong Guan. Sichuan Provincial CDC: Xianping Wu, Ningmei Zhang, Xiaofang Chen, Xuefeng Tang. Pengzhou CDC: Guojin Luo, Jianguo Li, Xiaofang Chen, Jian Wang, Jiaqiu Liu, Qiang Sun. Gansu Provincial CDC: Pengfei Ge, Xiaolan Ren, Caixia Dong. Maiji CDC: Hui Zhang, Enke Mao, Xiaoping Wang, Tao Wang. Henan Provincial CDC: Guohua Liu, Baoyu Zhu, Gang Zhou, Shixian Feng, Liang Chang, Lei Fan. Huixian CDC: Yulian Gao, Tianyou He, Li Jiang, Huarong Sun, Pan He, Chen Hu, Qiannan Lv, Xukui Zhang. Zhejiang Provincial CDC: Min Yu, Ruying Hu, Le Fang, Hao Wang. Tongxiang CDC: Yijian Qian, Chunmei Wang, Kaixue Xie, Lingli Chen, Yaxing Pan, Dongxia Pan. Hunan Provincial CDC: Yuelong Huang, Biyun Chen, Donghui Jin, Huilin Liu, Zhongxi Fu, Qiaohua Xu. Liuyang CDC: Xin Xu, Youping Xiong, Weifang Jia, Xianzhi Li, Libo Zhang, Zhe Qiu.
