# Supplementary material for: Breastfeeding and the Risk of Maternal Cardiovascular Disease: A Prospective Study of 300 000 Chinese Women
Source: J Am Heart Assoc. 2017 Jun 21;6(6):e006081. doi: 10.1161/JAHA.117.006081 (PMC5669201; doi:10.1161/JAHA.117.006081)

Supplemental Material

Table S1. Hazard ratios (95% confidence intervals) for cardiovascular disease associated with duration of breastfeeding among parous women who ever breastfed across different levels of adjustment

	Duration of breastfeeding per child, months					
	N events	>0-6 months	6-12 months	12-18 months	18-24 months	>24 months
**Model 1**						
All cardiovascular disease	47301	1.00 (0.96; 1.04)	0.98 (0.96; 1.00)	0.97 (0.96; 0.99)	0.95 (0.92; 0.97)	0.91 (0.88; 0.94)
Major cardiovascular disease	18348	1.00 (0.94; 1.07)	1.00 (0.97; 1.03)	1.00 (0.97; 1.02)	0.99 (0.96; 1.03)	0.94 (0.89; 0.98)
Fatal cardiovascular disease	3695	1.00 (0.84; 1.20)	0.96 (0.89; 1.03)	0.99 (0.94; 1.06)	0.95 (0.89; 1.02)	0.99 (0.91; 1.09)
Coronary heart disease	15825	1.00 (0.94; 1.07)	0.94 (0.91; 0.97)	0.90 (0.88; 0.93)	0.87 (0.83; 0.91)	0.82 (0.78; 0.87)
Stroke	22872	1.00 (0.94; 1.06)	0.98 (0.96; 1.01)	0.98 (0.95; 1.00)	0.97 (0.93; 1.00)	0.88 (0.84; 0.92)
Haemorrhagic stroke	2900	1.00 (0.82; 1.22)	1.04 (0.96; 1.12)	1.07 (1.00; 1.15)	1.11 (1.02; 1.20)	1.06 (0.95; 1.18)
Ischaemic stroke	13580	1.00 (0.93; 1.08)	1.01 (0.97; 1.04)	1.00 (0.97; 1.03)	0.99 (0.95; 1.04)	0.91 (0.86; 0.96)
**Model 2**						
All cardiovascular disease	47301	1.00 (0.96; 1.04)	0.97 (0.96; 0.99)	0.97 (0.96; 0.99)	0.94 (0.92; 0.96)	0.90 (0.87; 0.93)
Major cardiovascular disease	18348	1.00 (0.94; 1.07)	0.98 (0.95; 1.01)	0.97 (0.94; 1.00)	0.96 (0.93; 0.99)	0.90 (0.86; 0.95)
Fatal cardiovascular disease	3695	1.00 (0.84; 1.20)	0.89 (0.83; 0.96)	0.92 (0.87; 0.98)	0.87 (0.81; 0.94)	0.89 (0.82; 0.98)
Coronary heart disease	15825	1.00 (0.94; 1.07)	0.94 (0.91; 0.97)	0.92 (0.89; 0.94)	0.89 (0.85; 0.92)	0.84 (0.79; 0.89)
Stroke	22872	1.00 (0.94; 1.06)	0.97 (0.95; 1.00)	0.97 (0.95; 0.99)	0.96 (0.93; 0.99)	0.87 (0.83; 0.92)
Haemorrhagic stroke	2900	1.00 (0.82; 1.22)	1.02 (0.94; 1.10)	1.05 (0.98; 1.12)	1.08 (0.99; 1.17)	1.02 (0.91; 1.14)
Ischaemic stroke	13580	1.00 (0.93; 1.08)	0.99 (0.96; 1.03)	0.99 (0.96; 1.02)	0.98 (0.94; 1.02)	0.89 (0.84; 0.94)

**Model 3**						
All cardiovascular disease	47301	1.00 (0.96; 1.04)	0.97 (0.95; 0.99)	0.95 (0.94; 0.97)	0.93 (0.90; 0.95)	0.88 (0.85; 0.92)
Major cardiovascular disease	18348	1.00 (0.94; 1.07)	0.96 (0.93; 0.99)	0.95 (0.92; 0.97)	0.94 (0.91; 0.97)	0.88 (0.84; 0.92)
Fatal cardiovascular disease	3695	1.00 (0.84; 1.20)	0.88 (0.82; 0.95)	0.91 (0.86; 0.97)	0.86 (0.80; 0.92)	0.88 (0.80; 0.96)
Coronary heart disease	15825	1.00 (0.94; 1.07)	0.93 (0.90; 0.96)	0.89 (0.87; 0.92)	0.87 (0.84; 0.91)	0.82 (0.78; 0.87)
Stroke	22872	1.00 (0.94; 1.06)	0.96 (0.94; 0.98)	0.95 (0.92; 0.97)	0.94 (0.91; 0.97)	0.85 (0.81; 0.89)
Haemorrhagic stroke	2900	1.00 (0.82; 1.22)	0.99 (0.92; 1.07)	1.02 (0.95; 1.09)	1.04 (0.96; 1.13)	0.97 (0.87; 1.08)
Ischaemic stroke	13580	1.00 (0.93; 1.08)	0.97 (0.94; 1.00)	0.96 (0.93; 0.99)	0.95 (0.91; 0.99)	0.86 (0.81; 0.91)
**Model 4**						
All cardiovascular disease	47301	1.00 (0.96; 1.04)	0.96 (0.95; 0.98)	0.95 (0.93; 0.96)	0.92 (0.89; 0.94)	0.88 (0.85; 0.91)
Major cardiovascular disease	18348	1.00 (0.93; 1.07)	0.96 (0.93; 0.99)	0.94 (0.92; 0.97)	0.94 (0.90; 0.97)	0.88 (0.83; 0.92)
Fatal cardiovascular disease	3695	1.00 (0.84; 1.20)	0.88 (0.82; 0.94)	0.90 (0.85; 0.96)	0.85 (0.80; 0.91)	0.87 (0.79; 0.95)
Coronary heart disease	15825	1.00 (0.94; 1.07)	0.93 (0.90; 0.95)	0.89 (0.86; 0.91)	0.86 (0.83; 0.90)	0.81 (0.77; 0.86)
Stroke	22872	1.00 (0.94; 1.06)	0.96 (0.93; 0.98)	0.94 (0.92; 0.97)	0.93 (0.90; 0.96)	0.84 (0.80; 0.88)
Haemorrhagic stroke	2900	1.00 (0.82; 1.22)	0.99 (0.91; 1.07)	1.01 (0.94; 1.08)	1.03 (0.95; 1.12)	0.96 (0.86; 1.07)
Ischaemic stroke	13580	1.00 (0.93; 1.08)	0.97 (0.94; 1.00)	0.96 (0.93; 0.99)	0.94 (0.91; 0.99)	0.86 (0.81; 0.91)

Model 1: stratified by age at risk and study area; Model 2: Model 1 + adjustment for level of attained education, household income, smoking status, and alcohol use; Model 3: Model 2 + adjustment for systolic blood pressure, history of hypertension, physical activity, body mass index, and history of diabetes. Model 4 + adjustment for age at menarche, age at first birth, and total number of miscarriages, induced abortions, and stillbirths.

Table S2. Hazard ratios (95% confidence intervals) for cardiovascular disease associated with duration of breastfeeding per child among parous women across different levels of adjustment, using those who never breastfed as the reference group

		Duration of breastfeeding per child, months					
	**N events**	**Never**	**>0-6 months**	**6-12 months**	**12-18 months**	**18-24 months**	**>24 months**
**Model 1**							
All cardiovascular disease	48692	1.00 (0.95; 1.06)	0.99 (0.95; 1.03)	0.97 (0.95; 0.99)	0.96 (0.95; 0.98)	0.93 (0.91; 0.96)	0.90 (0.87; 0.93)
Major cardiovascular disease	18923	1.00 (0.92; 1.09)	0.90 (0.85; 0.97)	0.91 (0.88; 0.93)	0.90 (0.88; 0.93)	0.90 (0.86; 0.93)	0.85 (0.81; 0.89)
Fatal cardiovascular disease	3755	1.00 (0.77; 1.29)	1.00 (0.84; 1.20)	0.96 (0.90; 1.03)	1.00 (0.94; 1.06)	0.95 (0.89; 1.02)	1.00 (0.91; 1.09)
Coronary heart disease	16401	1.00 (0.92; 1.09)	0.97 (0.91; 1.04)	0.91 (0.89; 0.94)	0.88 (0.85; 0.91)	0.85 (0.81; 0.88)	0.80 (0.76; 0.85)
Stroke	23631	1.00 (0.93; 1.08)	0.95 (0.90; 1.00)	0.93 (0.91; 0.96)	0.93 (0.90; 0.95)	0.92 (0.89; 0.95)	0.84 (0.80; 0.88)
Haemorrhagic stroke	2956	1.00 (0.77; 1.31)	0.84 (0.69; 1.02)	0.87 (0.81; 0.94)	0.90 (0.84; 0.97)	0.93 (0.86; 1.01)	0.89 (0.79; 0.99)
Ischaemic stroke	14066	1.00 (0.91; 1.10)	0.89 (0.83; 0.96)	0.90 (0.87; 0.93)	0.89 (0.86; 0.92)	0.88 (0.85; 0.92)	0.81 (0.76; 0.85)
**Model 2**							
All cardiovascular disease	48692	1.00 (0.95; 1.06)	0.99 (0.95; 1.03)	0.97 (0.95; 0.98)	0.96 (0.95; 0.98)	0.93 (0.91; 0.96)	0.89 (0.86; 0.93)
Major cardiovascular disease	18923	1.00 (0.92; 1.09)	0.91 (0.85; 0.97)	0.90 (0.87; 0.92)	0.89 (0.86; 0.91)	0.88 (0.85; 0.91)	0.83 (0.79; 0.87)
Fatal cardiovascular disease	3755	1.00 (0.77; 1.29)	1.00 (0.84; 1.20)	0.89 (0.83; 0.95)	0.92 (0.86; 0.97)	0.87 (0.81; 0.93)	0.88 (0.80; 0.96)
Coronary heart disease	16401	1.00 (0.92; 1.09)	0.98 (0.92; 1.05)	0.92 (0.90; 0.95)	0.90 (0.87; 0.93)	0.87 (0.84; 0.91)	0.82 (0.78; 0.87)
Stroke	23631	1.00 (0.93; 1.08)	0.97 (0.91; 1.02)	0.93 (0.90; 0.95)	0.92 (0.89; 0.94)	0.91 (0.88; 0.94)	0.82 (0.78; 0.86)
Haemorrhagic stroke	2956	1.00 (0.77; 1.31)	0.84 (0.69; 1.02)	0.85 (0.79; 0.92)	0.87 (0.81; 0.93)	0.89 (0.82; 0.97)	0.84 (0.76; 0.94)
Ischaemic stroke	14066	1.00 (0.91; 1.10)	0.90 (0.83; 0.96)	0.89 (0.86; 0.92)	0.88 (0.85; 0.91)	0.87 (0.83; 0.91)	0.79 (0.75; 0.84)
**Model 3**							
All cardiovascular disease	48692	1.00 (0.95; 1.06)	1.00 (0.96; 1.04)	0.97 (0.96; 0.99)	0.96 (0.94; 0.97)	0.93 (0.91; 0.95)	0.89 (0.86; 0.92)
Major cardiovascular disease	18923	1.00 (0.92; 1.09)	0.93 (0.87; 0.99)	0.89 (0.87; 0.92)	0.88 (0.85; 0.90)	0.87 (0.84; 0.90)	0.81 (0.78; 0.85)
Fatal cardiovascular disease	3755	1.00 (0.77; 1.29)	1.01 (0.84; 1.20)	0.90 (0.84; 0.96)	0.91 (0.85; 0.96)	0.85 (0.80; 0.92)	0.89 (0.81; 0.97)
Coronary heart disease	16401	1.00 (0.92; 1.09)	0.99 (0.93; 1.06)	0.93 (0.90; 0.96)	0.89 (0.86; 0.92)	0.87 (0.83; 0.90)	0.82 (0.77; 0.87)
Stroke	23631	1.00 (0.93; 1.08)	0.95 (0.90; 1.01)	0.93 (0.90; 0.95)	0.92 (0.90; 0.94)	0.91 (0.88; 0.94)	0.83 (0.79; 0.87)
Haemorrhagic stroke	2956	1.00 (0.76; 1.31)	0.84 (0.69; 1.03)	0.84 (0.78; 0.90)	0.86 (0.80; 0.92)	0.88 (0.81; 0.95)	0.82 (0.73; 0.91)
Ischaemic stroke	14066	1.00 (0.91; 1.10)	0.92 (0.85; 0.99)	0.89 (0.86; 0.92)	0.88 (0.85; 0.91)	0.87 (0.83; 0.90)	0.78 (0.74; 0.83)

Adjustment as in Table S1

Table S3. Adjusted* hazard ratios (95% confidence intervals) for cardiovascular disease associated with total lifetime duration of breastfeeding among parous women

		Lifetime duration of breastfeeding, months					
	N events	Never	>0-12 months	12-24 months	24-36 months	36-48 months	>48 months
All cardiovascular disease	48692	1.00 (0.95; 1.06)	0.96 (0.93; 0.99)	0.97 (0.95; 0.99)	0.96 (0.94; 0.98)	0.92 (0.89; 0.94)	0.91 (0.88; 0.93)
Major cardiovascular disease	18923	1.00 (0.92; 1.09)	0.90 (0.86; 0.95)	0.91 (0.87; 0.94)	0.87 (0.84; 0.90)	0.80 (0.77; 0.83)	0.82 (0.78; 0.85)
Fatal cardiovascular disease	3755	1.00 (0.77; 1.29)	0.88 (0.74; 1.04)	0.98 (0.87; 1.09)	0.93 (0.85; 1.02)	0.81 (0.74; 0.89)	0.86 (0.79; 0.92)
Coronary heart disease	16401	1.00 (0.92; 1.09)	0.93 (0.89; 0.99)	0.92 (0.89; 0.96)	0.86 (0.83; 0.89)	0.86 (0.82; 0.90)	0.82 (0.79; 0.86)
Stroke	23631	1.00 (0.93; 1.08)	0.93 (0.89; 0.97)	0.93 (0.90; 0.96)	0.91 (0.88; 0.94)	0.86 (0.82; 0.89)	0.85 (0.82; 0.89)
Haemorrhagic stroke	2956	1.00 (0.76; 1.31)	0.84 (0.72; 0.99)	0.87 (0.78; 0.97)	0.81 (0.74; 0.89)	0.78 (0.70; 0.86)	0.84 (0.77; 0.93)
Ischaemic stroke	14066	1.00 (0.91; 1.10)	0.91 (0.85; 0.96)	0.89 (0.85; 0.93)	0.86 (0.83; 0.90)	0.80 (0.76; 0.84)	0.81 (0.77; 0.85)

*Analyses are stratified by age at risk and study area, and adjusted for level of attained education, household income, smoking status, alcohol use, systolic blood pressure, history of hypertension, physical activity, body mass index, history of diabetes, and number of livebirths.

Table S4. Adjusted* hazard ratios (95% confidence intervals) for cardiovascular disease associated with duration of breastfeeding among women with one livebirth, using those who never breastfed as the reference group

			Lifetime duration of breastfeeding, months					
	N events	Ever vs. never	Never	>0-6 months	6-12 months	12-18 months	18-24 months	>24 months
All cardiovascular disease	10760	0.95 (0.88; 1.01)	1.00 (0.95; 1.06)	0.96 (0.93; 0.99)	0.97 (0.95; 0.99)	0.96 (0.94; 0.98)	0.92 (0.89; 0.94)	0.91 (0.88; 0.93)
Major cardiovascular disease	3274	0.89 (0.80; 1.00)	1.00 (0.92; 1.09)	0.90 (0.86; 0.95)	0.91 (0.87; 0.94)	0.87 (0.84; 0.90)	0.80 (0.77; 0.83)	0.82 (0.78; 0.85)
Fatal cardiovascular disease	303	0.94 (0.62; 1.45)	1.00 (0.77; 1.29)	0.88 (0.74; 1.04)	0.98 (0.87; 1.09)	0.93 (0.85; 1.02)	0.81 (0.74; 0.89)	0.86 (0.79; 0.92)
Coronary heart disease	3597	0.89 (0.80; 0.99)	1.00 (0.92; 1.09)	0.93 (0.89; 0.99)	0.92 (0.89; 0.96)	0.86 (0.83; 0.89)	0.86 (0.82; 0.90)	0.82 (0.79; 0.86)
Stroke	4676	0.93 (0.84; 1.02)	1.00 (0.93; 1.08)	0.93 (0.89; 0.97)	0.93 (0.90; 0.96)	0.91 (0.88; 0.94)	0.86 (0.82; 0.89)	0.85 (0.82; 0.89)
Haemorrhagic stroke	350	0.92 (0.62; 1.37)	1.00 (0.76; 1.31)	0.84 (0.72; 0.99)	0.87 (0.78; 0.97)	0.81 (0.74; 0.89)	0.78 (0.70; 0.86)	0.84 (0.77; 0.93)
Ischaemic stroke	2689	0.90 (0.80; 1.02)	1.00 (0.91; 1.10)	0.91 (0.85; 0.96)	0.89 (0.85; 0.93)	0.86 (0.83; 0.90)	0.80 (0.76; 0.84)	0.81 (0.77; 0.85)

*Analyses are stratified by age at risk and study area, and adjusted for level of attained education, household income, smoking status, alcohol use, systolic blood pressure, history of hypertension, physical activity, body mass index, and history of diabetes.

Supplemental Figure Legends:

Figure S1: Adjusted* hazard ratios and 95% CIs for incident cardiovascular disease, coronary heart disease, and stroke associated with duration of breastfeeding per child among parous women who ever breastfed, by region.·*Analyses are stratified by age at risk and study area, and adjusted for level of attained education, household income, smoking status, alcohol use, systolic blood pressure, history of hypertension, physical activity, body mass index, and history of diabetes. The hazard ratios (HRs) are plotted on a floating absolute scale. Each square has an area inversely proportional to the standard error of the log risk. Vertical lines indicate the corresponding 95% confidence intervals (CIs).

Figure S2. Adjusted* hazard ratios and 95% CIs for incident cardiovascular disease, coronary heart disease, and stroke associated with duration of breastfeeding per child among parous women who ever breastfed, by birth cohort.*Analyses are stratified by age at risk and study area, and adjusted for level of attained education, household income, smoking status, alcohol use, systolic blood pressure, history of hypertension, physical activity, body mass index, and history of diabetes. The hazard ratios (HRs) are plotted on a floating absolute scale. Each square has an area inversely proportional to the standard error of the log risk. Vertical lines indicate the corresponding 95% confidence intervals (CIs).

Figure S1. Adjusted* hazard ratios for incident CVD, CHD, and stroke associated with duration of breastfeeding per child among parous women who ever breastfed, by region

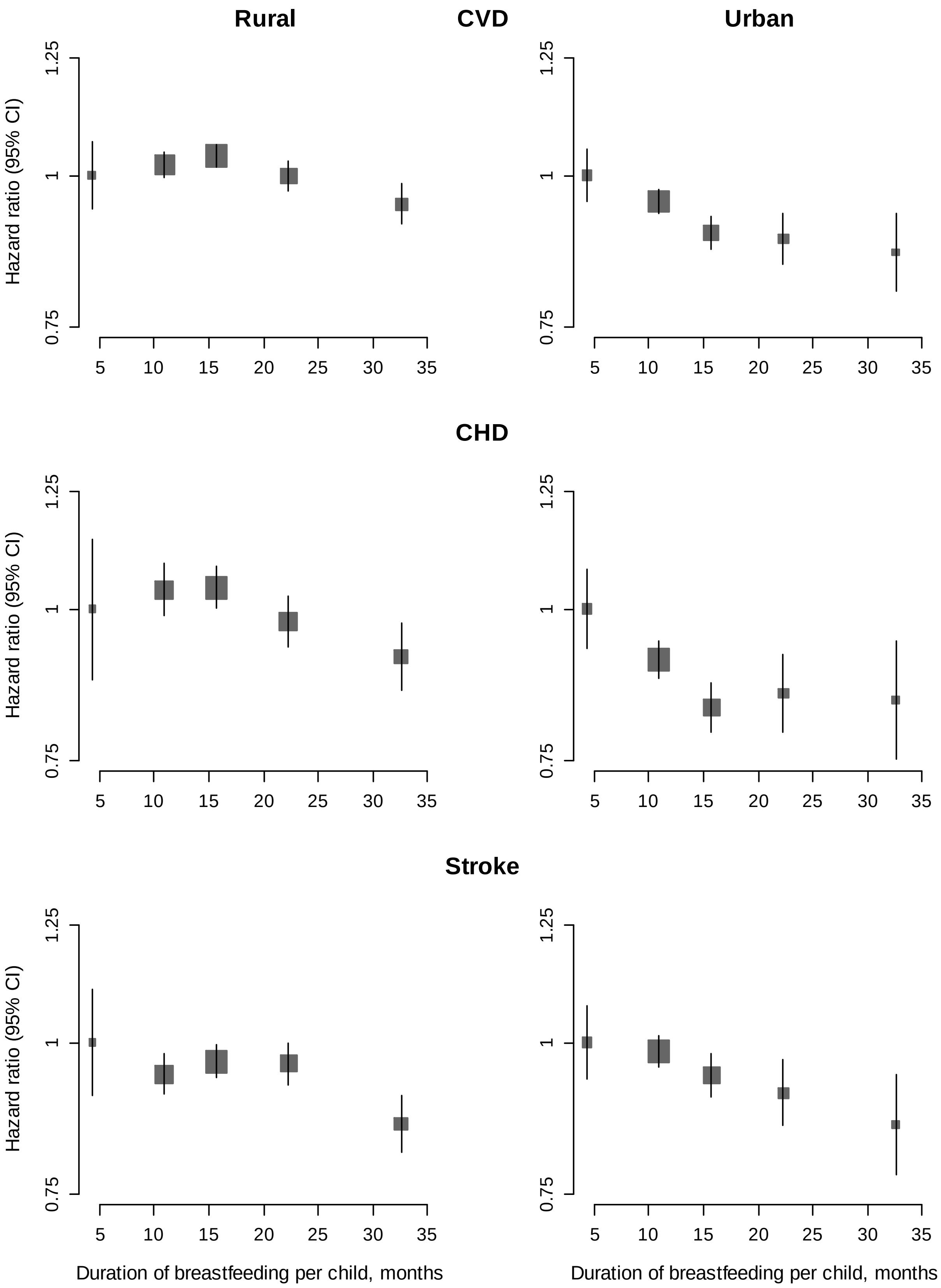

Figure S2. Adjusted* hazard ratios for incident CVD, CHD and stroke associated with duration of breastfeeding per child among parous women who ever breastfed, by birth cohort

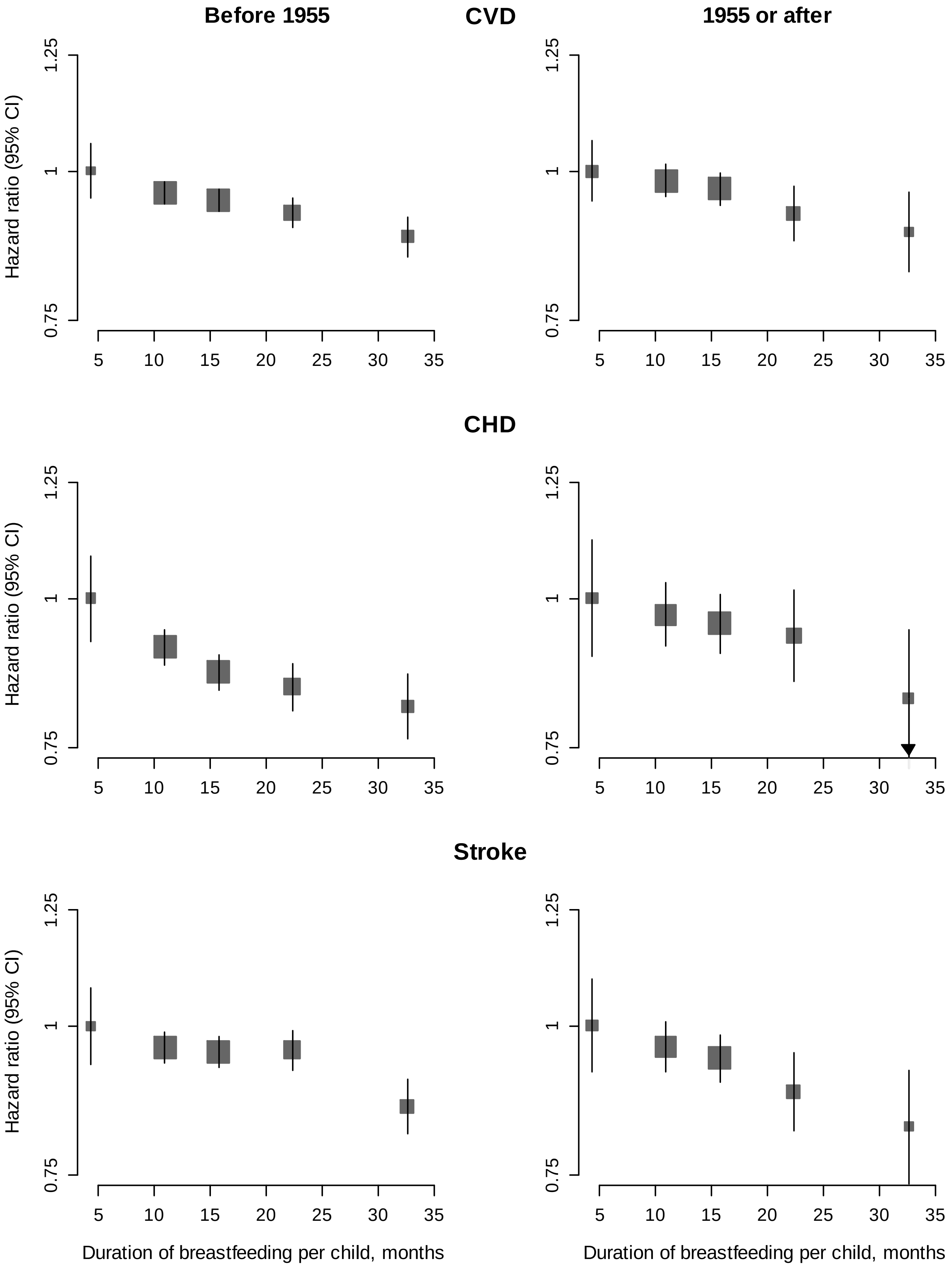

Supplement: Supplementary file 1 — Table S1. Hazard Ratios (95% CIs) for Cardiovascular Disease Associated With Duration of Breastfeeding Among Parous Women Who Ever Breastfed Across Different Levels of Adjustment Table S2. Hazard Ratios (95% CIs) for Cardiovascular Disease Associated With Duration of Breastfeeding Per Child Among Parous Women Across Different Levels of Adjustment, Using Those Who Never Breastfed as the Reference Group Table S3. Adjusted* Hazard Ratios (95% CIs) for Cardiovascular Disease Associated With Total Lifetime Duration of Breastfeeding Among Parous Women Table S4. Adjusted* Hazard Ratios (95% CIs) for Cardiovascular Disease Associated With Duration of Breastfeeding Among Women With One Livebirth, Using Those Who Never Breastfed as the Reference Group Figure S1. Adjusted* hazard ratios (HRs) and 95% CIs for incident cardiovascular disease, coronary heart disease, and stroke associated with duration of breastfeeding per child among parous women who ever breastfed, by region. *Analyses are stratified by age at risk and study area and adjusted for level of attained education, household income, smoking status, alcohol use, systolic blood pressure, history of hypertension, physical activity, body mass index, and history of diabetes mellitus. The HRs are plotted on a floating absolute scale. Each square has an area inversely proportional to the SE of the log risk. Vertical lines indicate the corresponding 95% CIs. Figure S2. Adjusted* hazard ratios (HRs) and 95% CIs for incident cardiovascular disease, coronary heart disease, and stroke associated with duration of breastfeeding per child among parous women who ever breastfed, by birth cohort. *Analyses are stratified by age at risk and study area and adjusted for level of attained education, household income, smoking status, alcohol use, systolic blood pressure, history of hypertension, physical activity, body mass index, and history of diabetes mellitus. The HRs are plotted on a floating absolute scale. Each square has an area inv [file JAH3-6-e006081-s001.pdf]
